# A review on chest CT scanning parameters implemented in COVID-19 patients: bringing low-dose CT protocols into play

**DOI:** 10.1186/s43055-020-00400-1

**Published:** 2021-01-05

**Authors:** Javid Azadbakht, Daryoush Khoramian, Zahra Sadat Lajevardi, Fateme Elikaii, Amir Hossein Aflatoonian, Bagher Farhood, Masoud Najafi, Hamed Bagheri

**Affiliations:** 1 Department of Radiology, Faculty of Medicine, Kashan University of Medical Sciences, Kashan, Iran; 2 The Advocate Center for Clinical Research, Ayatollah Yasrebi Hospital, Kashan, Iran; 3 Faculty of Medicine, Kashan University of Medical Sciences, Kashan, Iran; 4 Department of Medical Physics and Radiology, Kashan University of Medical Sciences, Kashan, Iran; 5 Radiology and Nuclear Medicine Department, School of Paramedical Sciences, Kermanshah University of Medical Sciences, Kermanshah, Iran; 6 Radiation Sciences Research Center (RSRC), AJA University of Medical Sciences, Tehran, Iran

**Keywords:** COVID-19 pneumonia, Computed tomography, Radiation dose, Low dose

## Abstract

**Background:**

This study aims to review chest computed tomography (CT) scanning parameters which are utilized to evaluate patients for COVID-19-induced pneumonia. Also, some of radiation dose reduction techniques in CT would be mentioned, because using these techniques or low-dose protocol can decrease the radiation burden on the population.

**Main body:**

Chest CT scan can play a key diagnostic role in COVID-19 patients. Additionally, it can be useful to monitor imaging changes during treatment. However, CT scan overuse during the COVID-19 pandemic raises concerns about radiation-induced adverse effects, both in patients and healthcare workers.

**Conclusion:**

By evaluating the CT scanning parameters used in several studies, one can find the necessity for optimizing these parameters. It has been found that chest CT scan taken using low-dose CT protocol is a reliable diagnostic tool to detect COVID-19 pneumonia in daily practice. Moreover, the low-dose chest CT protocol results in a remarkable reduction (up to 89%) in the radiation dose compared to the standard-dose protocol, not lowering diagnostic accuracy of COVID-19-induced pneumonia in CT images. Therefore, its employment in the era of the COVID-19 pandemic is highly recommended.

## Background

New coronavirus (commonly known as COVID-19) pneumonia, which was first reported in Wuhan, Hubei Province, China, in December 2019, followed rapid spread across China and worldwide [1]. This virus is highly contagious and can be spread from person to person by either an infected person or an asymptomatic carrier. The virus can spread in communities and cross borders rapidly. Respiratory droplets (in most of the cases), close contact, and transmission via the digestive tract are possible routes of transmission [2, 3]. Thus far, no curative drug or effective vaccine for COVID-19 is available; therefore, diagnosing the disease at an early stage and to quarantine the infected patients are the most rational approaches to alleviate. China government has published a guideline on Diagnosis and Treatment of Pneumonitis Caused by 2019-nCoV; its latest version says reverse transcription-polymerase chain reaction (RT-PCR) or gene sequencing for samples extracted from the respiratory tract or blood is needed to confirm the diagnosis of COVID-19, which in turn is the main indicator of the need for hospitalization [4]. Though, limitations related to kit performance, sample extraction and sample transportation resulted in about 30 to 60% of RT-PCR tests done on throat swab samples to be positive at initial presentation. This lack of sensitivity will cause many COVID-19 patients not to be identified, leaving a larger population at risk of infection [5].

Imaging also plays a crucial role in the follow-up of novel coronavirus-infected pneumonia (NCIP) patients. Computed tomography (CT) is regarded as the preferable imaging modality in clinically suspected patients and is advantageous to monitor patients during treatment [6, 7]. This imaging modality potentially can spot patients who are highly suspicious of NCIP, regarding clinical conditions with false-negative RT-PCR results [8, 9]. The findings obtained from the CT scan may also provide information on the disease severity [10–12]. Moreover, considering similarities in diagnostic patterns of most viral pneumonia, it has been reported in many recent studies that the information derived from CT might be helpful to discriminate COVID-19 from other pathogens causing pneumonia [13]. Chest CT scan (about 56–98% sensitive in COVID-19 detection) is relatively easy to perform and let clinicians diagnose pneumonia fast enough to make important therapeutic decisions effectively early in the disease process [11, 14].

The ionizing radiation exploited in medical imaging is one of the main sources of radiation exposure. It has been reported that the growing popularity and usage of imaging modalities which put ionizing radiation to use leads to an increase in cancer risk [15]. According to the linear no-threshold (LNT) model, there is a direct relationship between the incidence of solid cancers and ionizing radiation, even at low doses. The LNT states that any minimal amount of radiation exposure can increase the incidence of mutation or cancer [16, 17]. The number and dose of CT studies are one of the largest sources of imaging exposure. A typical chest CT scanning delivers a dose of 4–7 mSv to the patient [18], and it raises the risk of cancer incidence and mortality [19, 20]. Thus, although CT scan has played an important role in the management of COVID-19-infected patients, it can promote malignancy. Given that CT scans are mainly performed in patients with clinical suspicion on COVID-19 pneumonia as a standard method to make decisions on hospitalization or discharge, it is possible for each patient suspicious of COVID-19 to undergo one (or more) CT scan(s). Due to the large and uprising number of COVID-19-infected patients worldwide, the risk of developing various types of cancers is worrisome [18]. Considering the growing number of chest CT scans requested by referring physicians and the necessity of decreasing the potential risks posed on patients by ionizing radiation, a dose reduction protocol is an essential requirement [21]. Following the ALARA (as low as reasonably achievable), recommended by the International Commission of Radiological Protection (ICRP), during the daily practice of radiology is necessary, even in the setting of pandemic events [22]. For that reason, we should give thought to optimizing CT protocols implemented in COVID-19-infected patients, to consequently expect a reduction in the patient’s radiation dose.

In this study, we will cover the following sections: (1) chest CT scanning parameters employed so far in patients with COVID-19 infection, (2) CT scanning parameters which can affect the radiation dose (such as scan length, tube current, tube potential, pitch factor, etc.), and (3) low-dose chest CT protocols. We will also review the studies featuring low-dose CT protocols for chest imaging to detect the COVID-19 pneumonia.

## Main text

### Chest CT scanning parameters used in the diagnosis of COVID-19 infection

Some studies have reported the chest CT findings of the patients infected with COVID-19. In some of these studies, CT scanning parameters have been mentioned, which are extracted and enlisted in Table 1.

**Table 1:** The data extracted from a number of studies conducted on patients infected with COVID-19: focusing on chest CT scanning parameters

Author name	No. of cases	Studied population	Tube voltage (kV)	Tube current (mA)	Tube current-time (mAs)	Pitch factor	CTDIvol (mGy)	DLP (mGy cm)
Yoon et al. [23]	9	Median age, 54 years	120	–	30 (low dose) to 60–120 (standard)	–	–	–
Xu et al. [24]	90	Adults (18–86 years)	120	210	–	–	–	–
Xia et al. [25]	20	Pediatrics (1 day–14 years 7 months)	120	100 to 150	–	1	–	–
Wang et al. [26]	114	Adults (23–78)	120	320	–	1–1.5 (for severe and critical cases)	–	–
Li et al. [27]	–	–	120	–	100–250	1–1.5	–	–
Liu et al. [28]	59	Pregnant women (*n* = 41) and non-pregnant adults (*n* = 14) (22–42 years) and pediatrics (2 months–9 years, *n* = 4)	120 (pregnant women and non-pregnant adults) and 100 (pediatrics)	10–300 (pregnant women), 120–380 (non-pregnant adults), and 30–100 (pediatrics)	–	1.375:1 (pregnant women), 1.0875:1 or 1.375:1 (non-pregnant adults), and 0.969:1 (pediatrics)	–	50–150 (pregnant adults)
Shen et al. [29]	44	Mean age for the non-severe patient group, 52.17 ± 12.55 years and for the severe patient group, 57.25 ± 13.38 years	120	30–140	–	–	–	–
Song et al. [12]	51	Adults (16–76 years)	120	180–400	–	1.5	–	–
Xu et al. [30]	50	Pediatrics (*n* = 5) and adults (*n* = 45) (3–85 years)	120	40–250	–	0.984:1	–	–
Albarello et al. [31]	2	Adults (66 and 67 years)	120	250	–	1.375	–	–
Pan et al. [32]	21	Adults (25–63 years)	120	–	–	–	8.4 ± 2.0 (5.2–12.6)	–

As shown in Table 1, tube voltage is the same in all adult studies (120 kV). Other scanning parameters (such as tube current, pitch factor, and slice thickness) differ from one study to another. In a study by Wang et al. [26], chest CT features of confirmed COVID-19 patients were evaluated, in Xiaogan, Hubei, China. In that study, all patients underwent chest CT examination with a tube current of 320 mA, tube voltage of 120 kV, pitch factor of 1–1.5 (for severe and critical cases), and collimator width of 0.5–1.5 mm. In conclusion, they mentioned that a spiral CT scan, as a sensitive diagnostic tool, can be used to detect early disease and assess its progression. Also, they reported that the spiral chest CT scan is more accurate and sensitive compared to nucleic acid detection in COVID-19 diagnosis [26]. In another study, Song et al. [12] investigated chest CT findings of confirmed COVID-19 patients using RT-PCR in Shanghai, China. CT scanning parameters in their study were as follows: tube voltage of 120 kV, tube current of 180–400 mA, section thickness of 5 mm, and pitch factor of 1.5 [12]. Shen et al. [29] validated the performance of a developed computer tool in order to quantitatively evaluate COVID-19 pneumonia using CT images by comparing the data obtained from computer and radiologist’s notes. In that study, CT scanning parameters were as follows: tube voltage of 120 kV, tube current 30–140 mA, and slice thickness of 0.625 and 1 mm [29]. As mentioned earlier, one of the CT scanning parameters that varies remarkably between different studies is the current tube, which can affect the amount of dose absorbed by the patient (described in detail in the next section).

Moreover, the CT scanning parameters used to diagnose the COVID-19 infection can be different, depending on the studied population. For example, Liu et al. [28] assessed CT features in pregnant women, non-pregnant adults, and children with COVID-19 pneumonia. In that study, the tube voltage used for pregnant women and non-pregnant adults was 120 kV, but it was 100 kV for children. Also, automatic tube current used for pregnant women, non-pregnant adults, and children were 10–300, 120–380, and 30–100 mA, respectively. Helical pitch factor for pregnant women, non-pregnant adults, and children were 1.0875:1 or 1.375:1, 1.375:1, and 0.969:1, respectively [28]. In another study, Xia et al. [25] investigated chest CT findings in pediatric patients with COVID-19 infection. Reported CT scanning parameters in that study were as follows: tube voltage of 12.0 kV, tube current of 100 to 150 mA, collimation of 0.6 mm, and pitch factor of 1:1. As stated in latter two studies [25, 28], the value of scanning parameters used for pregnant women and children were quantitatively lesser than those of adults, an effort to reduce the radiation dose in these age groups. Regarding the radiosensitivity of fetuses and pediatric patients, attention should be drawn to radiation protection principles and dose reduction techniques in CT.

### Radiation dose reduction techniques in computed tomography

Before assessment of the scanning parameters affecting the radiation dose in CT, it is necessary to introduce the dose indices in the CT scan.

The primary dose descriptor for CT is computed tomography dose index (CTDI). The CTDI describes the summation of all dose contributions along the *z*-axis [33, 34]:

1 $$ {\mathrm{CTDI}}_{100}=\frac{1}{N.T}.{\int}_{-50\  mm}^{+50\  mm}D(z). dz $$

where *D*(*z*) is the value of the dose at a given location, *z*, and N.T is the nominal beam width [34, 35]. The CTDI_100_ is measured in the center (CTDI_100c_) and periphery (CTDI_100p_) of a polymethyl methacrylate (PMMA) phantom with an ionization chamber with an active length of 100 mm.

Other dose indices for CT dosimetry are weighted CTDI (CTDIw), volumetric CTDI (CTDIv), and dose length product (DLP). The DLP indicates the total amount of radiation received by the patient in a CT examination. The DLP is directly related to the CTDIv (mGy) and length of the scan area (cm) along the longitudinal axis (*z*-axis) and is measured in milli-gray × cm (mGy × cm). It eases comparison between the amount of radiation exposure in similar examinations [36]. Table 2 presents mathematical expressions of these indices which are well-defined in almost all CT scanners.

**Table 2:** Common CT dose descriptors

Dose index	Mathematical formula
Weighted CTDI [37]	CTDIw (mGy) = 1/3 CTDI_100,center_ + 2/3 CTDI_100,periphery_
Volumetric CTDI [37]	CTDIv (mGy) = CTDIw/pitch
DLP [38]	CTDIv × scan length (mGy × cm)

*mGy* milli-Gray, *pitch* the ratio of the table increment per rotation to the nominal beam width

Different organs respond differently to radiation. Given the same amount of radiation, different damages in different organs will ensue. Effective dose measures biologic tissue sensitivity to ionizing radiation effects and is expressed in millisievert. It is the summation of weighted organ dose values (*D*_T,R_) and is calculated as follows [39]:

2 $$ E=\sum {w}_{\mathrm{T}}\sum {w}_{\mathrm{R}}{D}_{\mathrm{T},\mathrm{R}} $$

where *W*_R_ is the radiation weighting value and *W*_T_ is the tissue weighting factor. For diagnostic imaging X-rays, *W*_R_ is 1. First time, tissue weighting factors were introduced in ICRP report 26. The tissue weighting factors had been updated two times in ICRP 60 and ICRP 103 reports [35].

#### CT scanning parameters affecting the radiation dose

There are multiple physical and technical dose reduction strategies to reduce radiation dose in CT imaging. Of those, we will review some scanning parameters and equipment-related factors here. Table 3 represents some equipment-related and application-related factors affecting radiation dose in CT (for further details, the reader can refer to Tack et al. [34]).

**Table 3:** Factors affecting radiation dose in CT [34]

Equipment-related factors	Application-related factors
Beam filtration Beam shaper Beam collimation Detector array Data acquisition system Spiral interpolation Adaptive filtration Overranging Automatic exposure control (AEC) system Reconstruction method	Scan parameters • Tube current-time (mAs) • Tube potential (kVp) • Slice collimation and slice thickness • Pitch • Object diameter of patient weight Examination parameters • Scan length • Number of scan series • Number of rotations in dynamic CT studies

##### Scan length

Optimization of patient positioning and scan length adjustment avoids radiation to tissues beyond the region of interest and thereby decreases the absorbed dose without any adverse effect on the image quality [40, 41]. As indicated in Table 2, the DLP is directly proportional to scan length. Hence, the patient dose can be decreased by reducing scan length.

##### Tube current

Adjusting the tube current (mA) is another measure, which results in reduced radiation in the CT scan [42]. The tube current is in proportion to the number of electrons moving from the cathode to anode and thereby is proportional to the quantity of the X-ray emitted from the anode. With tube current increasing, the radiation dose increases linearly. For example, 50% reduction in tube current leads to a 50% reduction in radiation dose [43]. Zarb et al. [44] reported a dose reduction of 20–33%, with a 20–33% reduction in the tube current. Reid et al. [45] measured radiation dose at different tube current-time (mAs) (50–400 mAs) levels in phantom. They also confirmed a linear relationship between tube current and radiation dose.

On the other hand, reducing mAs (proportional to the number of photons absorbed by the detector) leads to decreased image quantum noise (*σ*) [46]. Image noise is inversely proportional to the square root of mAs:

3 $$ \sigma =1/\sqrt{\mathrm{mAs}} $$

It is noteworthy that in order to reduce CT image noise, some methods such as the use of iterative reconstruction algorithms and image filters are proposed which would be discussed later.

##### Automatic exposure control

Automatic exposure control (AEC) is growing in popularity as an important function in CT scanners, the same exploit as it functions in radiograph machines and has been promising for radiation dose reduction [47]. The main principle of the AEC system is to optimize CT examinations through adapting the tube current to the patient’s size, shape, and attenuation [48]. In CT scanners, the AEC system makes programmed and dynamic tube current adjustment possible, which in turn preserves image quality between patients and for a single patient [49]. The modulation of the tube current can occur in the *X*-*Y* plane (angular modulation), along the *Z*-direction (longitudinal modulation), and in the *X*-*Y*-*Z* plane (combined modulation) [50, 51]. Gudjonsdottir et al. [52] reported that the proper use of AEC can decrease radiation dose values by 15 to 40%. Singh et al. [53] reported 50 to 75% radiation dose reduction could be achieved with the combined modulation technique in chest and abdomen CT in children.

##### Tube potential

Tube potential is the peak potential (kVp) applied to the X-ray tube, which reflects the energy of the X-ray photons. It has been proven that reduction of the kVp will result in the reduction of the radiation dose. In contrary to the tube current, the tube potential does not linearly correlate with the radiation dose [42, 54]. Huda et al. [54] reported the output values of 1.5 at 100 kVp, 2.5 at 120 kVp, and 3.4 at 140 kVp compared to the 80 kVp. Hamberg et al. [55] measured CTDIw at 80, 100, 120, and 140 kVp and stated that absorbed radiation dose increases as tube voltage to the power of 2.5 ± 0.1 in head mode and 2.8 ± 0.1 in body mode. Zarb et al. [44] examined the effect of reducing the tube potential on radiation dose and image quality parameters for head, abdomen, and chest CT examinations in four CT scanners. They reported that a 14–17% reduction in kVp can result in a 32–38% dose reduction; for instance, by 15% reduction of the kVp (130 to 110 kVp), the CTDI reduces by 38% (7.56 to 4.69 mGy). Kubo et al. [40] reviewed dose reduction strategies in chest CT and concluded that using lower kVp is particularly useful for pediatric patients and small size adults weighing less than 55 kg. They suggested taking advantage from the Care KV, provided by the Siemens for the tube voltage automatic selection, to overcome this problem.

##### Pitch factor

Pitch factor (simply pitch) is defined as the ratio of the CT table increment distance during one gantry rotation to the total beam width. For single-slice CT scanners, increasing the pitch leads to a decrease in the radiation dose proportionally. Mahesh et al. [56] reported radiation dose values of 12.72, 6.68, and 3.62 mGy for pitch factors of 0.5, 1, and 2, respectively. In contrast, for multi-slice helical CT scanners, radiation dose is independent of pitch factor. They reported radiation dose values of 9.92, 9.94, and 10.12 mGy for pitch factors of 2, 4, and 8, respectively. An explanation for this is the conception of effective mAs, so that the effective mAs or mAs per slice is the actual mAs value divided by the pitch (effective mAs = mAs/pitch) [57]. As the pitch increases in multi-slice CT scanners, the noise will increase with pitch and, subsequently, the CT scanner automatically increases mAs (to keep constant effective mAs) to prevent image quality degradation. This type of adjustment is available almost in all multi-slice CT scanners [34]. If one applies constant mAs, the radiation dose will decrease with increasing the pitch factor. Therefore, the CT users must gain enough knowledge on CT scanners they are working with and beware of the differences between mA and effective mA.

##### Reconstruction algorithm and image filter

Dose reduction in CT can be achieved by reducing the tube current (mA) or tube potential (kVp) or combination of both. Both of these methods will increase image noise. Iterative image reconstruction methods have been introduced in order to decrease the image noise in CT images. Yanagawa et al. [58] investigated the effects of adaptive statistical iterative reconstruction (ASIR) method on the computer-aided detection (CAD) system in pulmonary nodules using routine CT protocol and low-dose CT. They investigated 34 patients on 64-slice multi-detector CT scanner with 120 kVp at 100 mAs (routine CT protocol) and automatically adjusted mA (lower dose CT) with three different combinations of filtered back-projection and ASIR algorithms: 0% (pure filtered back-projection), 50%, and 100% (pure ASIR). The images were reviewed by three radiologists, and their results showed a significantly higher CAD sensitivity at 100% ASIR algorithm compared to 0% ASIR algorithm. Also, the effective dose was significantly higher in routine CT protocol compared to lower dose CT (10.77 mSv vs. 2.67 mSv) [58].

Another way of image noise reduction is using image filters (or kernels). Image filters can be applied on raw data or the reconstructed images. In a phantom study, Khoramian et al. [59] investigated the effect of different reconstruction kernels on noise and spatial resolution of CT images. In that study, an image was obtained from a conventional quality control phantom and it was reconstructed with five different kernels on Siemens Emotion 6 CT scanner. The employed kernels were H10, H30, H40, H50, and H70 (“H” indicates the scanned region, i.e., the head, and the numbers represent the softness and sharpness of the kernels, as H10 and H70 are the softest and the sharpest filters, respectively). Their findings indicated that the image noise and the spatial resolution are influenced by the kernel, as from H10 to H70, the image noise increases from 2.61 to 19.59, while the spatial resolution will get improved from 1.80 to 1.04 mm [59].

#### Low-dose CT protocol

There is no quantitative well-defined low-dose CT protocol. However, the aim of this protocol is to reduce the radiation dose delivered to the patient by changing radiation parameters mainly kVp and mAs. In fact, the main goal of low-dose CT protocol is to achieve acceptable diagnostic accuracy while keeping radiation dose at lower levels. One of the first efforts in performing low-dose chest CT was done by Naidich et al. [60] in 1990. They investigated and compared chest CT images of 12 patients between standard protocol and low-dose CT. Among study cases, two were normal and ten cases had evidence of diffuse lung disease. Two CT protocols were used and the images were evaluated independently by two experienced chest radiologists. The first set of CT scan images was obtained with 140 mA, 120 kV, 2-s scan time, 10 mm thickness, and standard construction algorithm. Low-dose protocol differed from the standard protocol only in tube current (10 mA). In conclusion, they reported that visualization of parenchymal structures was not affected by reducing mA [60].

Rusinek et al. [61] assessed the effectiveness of low-dose CT in the identification of pulmonary nodules. Six radiologists with 1 to 16 years of experience reviewed 864 (432 taken with the low-dose protocol and 432 taken with the conventional protocol) positive images and rated a 4-point scale for each image. Conventional scans were obtained at 120 kVp and 200 mAs with a slice thickness of 10 mm. The low-dose images were acquired with 20 mAs but all other scan parameters were the same as conventional images. Sensitivities of conventional and low-dose CT images were 63% and 60%, respectively. Also, specificities of conventional and low-dose CT images were 91% and 88%, respectively. There were no significant differences between sensitivity and specificity of low-dose and conventional images. Finally, they conclude that the low-dose CT protocol is acceptable for pulmonary nodule identification [61]. Rogalla et al. [62] examined chest low-dose CT examination in pediatric patients done for different indications. Tube voltage of 120 kV and mAs values of 12.5 to 75 per slice were applied. In that study, the diagnostic value of low-dose CT examinations was compared to standard-dose chest CT examinations of adults (tube voltage of 120 kV and 175 mAs per slice) in terms of technical image quality (noise and artifacts). Their results showed that for all indications in the chest CT scanning of children, the low-dose protocol provides sufficient image quality, without loss of diagnostic data. Also, the radiation dose obtained from the low-dose CT protocol was approximately 5–20% of a standard-dose CT protocol [62]. Ono et al. [63] compare image quality and radiation dose between low-dose CT and follow-up standard CT protocols for lung cancer screening in 19 adult patients. Low-dose CT images were obtained with 120 kVp, 30–50 mA, total beam collimation of 16 mm, and pitch of 1.438. Follow-up CT images were taken with 120 kVp, AEC (max 150 mAs), total beam collimation of 16 mm, and pitch of 1.188. There were no significant differences between these two protocols (low-dose CT and follow-up CT) for diagnosing lung cancer. Moreover, effective dose values ranged between 1.3 and 3.4 mSv and 8.5 and 14.0 mSv for low-dose and follow-up CT protocols, respectively. In conclusion, they suggested that low-dose CT can be used as an alternative to follow-up standard CT, an attempt to reduce radiation dose [63]. Kubo et al. [64] compared CT images of 59 patients with two different CT protocols: low-dose CT with 50 mAs at 120 kVp and 150 mAs at 120 kVp (other CT scanning parameters were identical). They found that CTDIw in low-dose CT is significantly lower than that of standard CT (5.36 mGy against 16.1 mGy). On the other hand, there was no significant difference in the deviation of the readers between low-dose CT and standard-dose CT [64].

## Perspective of future research

The first patient infected with a novel coronavirus was reported in 2019 December, Wuhan, China, and in less than 6 months (as announced by the World Health Organization), the total number of COVID-19 cases worldwide has crossed 5 million (https://www.who.int/emergencies/diseases/novel-coronavirus-2019/situation-reports)*.*

CT scan role has been approved as a diagnostic tool to help in evaluation, diagnosis, and treatment management in COVID-19-infected patients. Despite the mentioned advantages of chest CT scan, and its uses in making decisions for infected patients hospitalization or discharge, CT scan overuse raises concerns on increased radiation.

According to the LNT model, there is no safe level of radiation exposure and any radiation dose greater than 0 can lead to cancer or genetic mutations [16]. Hence, in X-ray-based medical imaging modalities, reduction in patient’s radiation, with or without negligible change in the image quality, is important [65]. In this regard, following as low as reasonably achievable (ALARA) principles can play a key role in radiation protection [46].

Based on the studies mentioned above, the scanning parameters (such as tube current and tube voltage) which are used to diagnose the COVID-19 pneumonia on CT scan can be optimized pursuing low-dose CT protocols. However, dosimetric indicators (such as CTDI, DLP, effective dose, etc.) were not mentioned in most of the studies and we could not discuss these quantities further. In a recent study by Dangis et al. [66], the accuracy and reproducibility of the low-dose chest CT protocol to diagnose COVID-19 pneumonia were evaluated. Mentioned CT scanning parameters were as follows: tube voltage of 100 kVp, tube current-time of 20 mAs, pitch factor of 1.2, and 0.5 second gantry rotation time. The mean effective radiation dose obtained from the low-dose protocol was submillisievert (0.56 ± 0.25 mSv). Finally, they concluded that low-dose submillisievert chest CT scan can be applied to assess COVID-19 pneumonia rapidly, accurately, and reproducibly [66]. Additionally, Radpour et al. [21] recommended CT scanning parameters of kVp 100–120, mAs 50–100, pitch factor 0.8–1.5, and thickness 1–3 mm, in order to minimize the radiation dose of patients. In conclusion, they stated that a low-dose high-resolution CT protocol might be more advantageous than RT-PCR, particularly in highly infected societies with low availability of PCR kits [21]. Kang et al. [67] also recommended the low-dose CT protocol in the diagnosis and management of COVID-19-induced pneumonia. They evaluated COVID-19 pneumonia in two adults of similar age, height, and body size by standard-dose and low-dose protocols and reported that the image quality in both protocols was excellent; nevertheless, effective dose and DLP values in the low-dose protocol were 0.203 mSv and 14.5 mGy cm, respectively, compared with 1.8074 mSv and 129.1 mGy cm in the standard-dose protocol [67]. In another report, Tofighi et al. [68] represented that chest CT low-dose protocol compared to conventional CT chest protocol reduced radiation dose to less than 50% without a remarkable impact on the diagnostic value. Shiri et al. [69] proposed a low-dose protocol based on deep learning algorithms and reported that using this protocol (kVp 90, mAs 20–40, pitch factor 0.75) compared to full-dose CT protocol (kVp 100–120, mAs 100–150, pitch factor 1.3–1.8) reduced CTDIvol, as a good dosemetric for comparing the effective dose and radiation-induced risks, by up to 89% which reflects a considerable decrease of the radiation dose value associated with CT examinations [69].

**Fig. 1:**
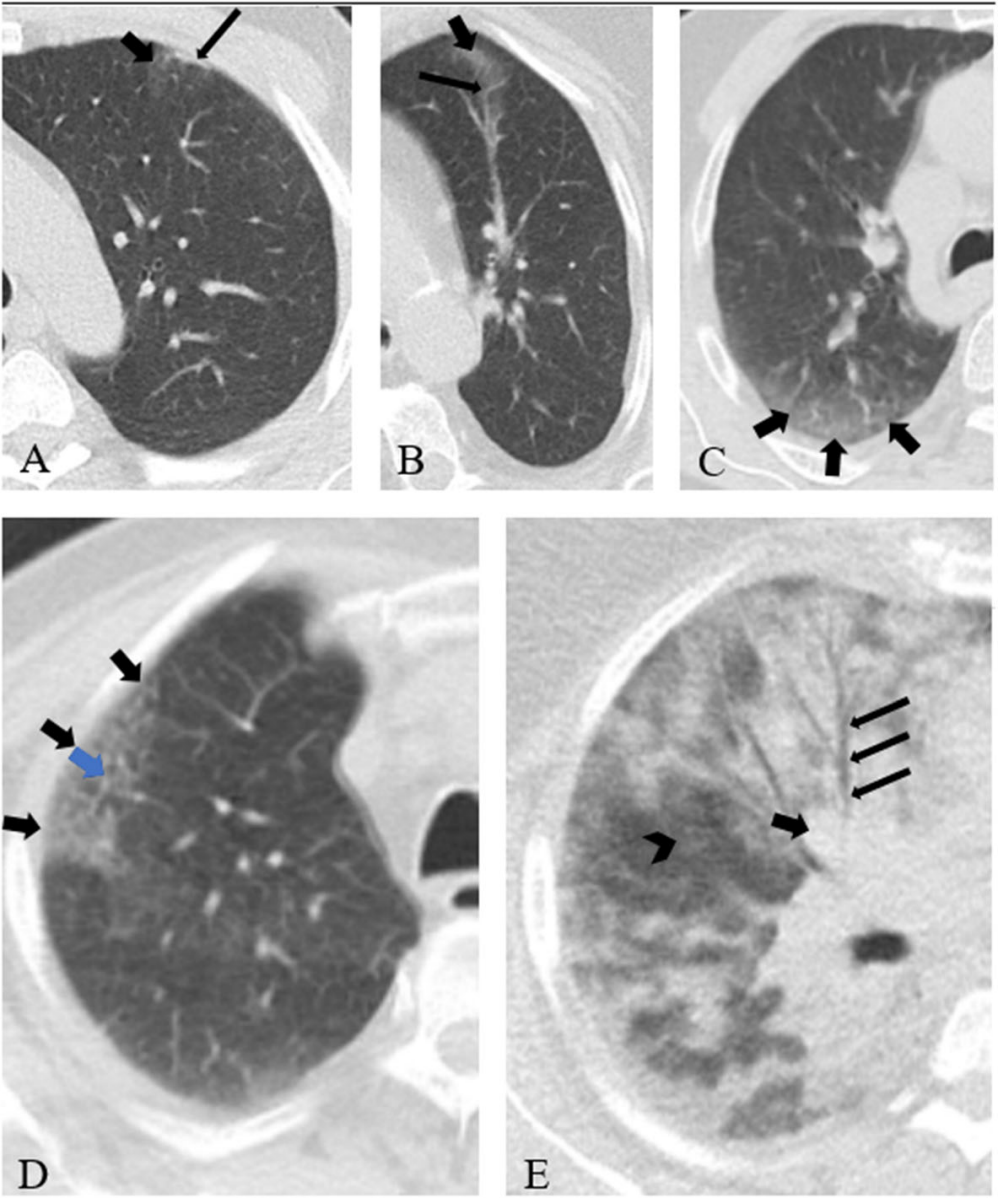
Low-dose chest CT scan images in 5 different patients with RT-PCR-confirmed COVID-19 pneumonia, showing a variety of diseased lung extension and density, comparable with the standard-dose CT scan. **a** Peripheral area of ground-glass opacity (thick short arrow) with overlying pleural thickening (thin long arrow). **b** Mixed central-peripheral lesion of ground-glass opacity (thick short arrow) with a prominent intralesional vessel (thin long arrow). **c** Peripheral area of ground-glass opacity (black arrows) with prominent intralesional vessels. **d** Peripheral area of crazy paving pattern (black arrows) with thickened interlobular septa within (blue arrow). **e** Mixed ground-glass opacity (arrowhead) and consolidation (thick black arrow). Long thin arrows point out air-bronchogram [images are from first author’s institution, implementing low-dose CT scan protocol, with scanning parameters as follows: kVp 120, mAs 50, pitch factor 1, thickness 3 mm]

From a clinical point of view, recent findings on evaluation of ground-glass and consolidative opacities (as the main criteria in diagnosing COVID-19 pneumonia) showed comparable results for both low-dose and standard-dose CT chest protocols [68]. Also, low-dose chest CT scan images in 5 different patients with RT-PCR-confirmed COVID-19 pneumonia (from our institution [Shahid Beheshti Hospital, Kashan, Iran]; CT scanning parameters: kVp 120, mAs 50, pitch factor 1, thickness 3 mm) show that variety of diseased lung extension and density are comparable with the standard-dose CT scan (Fig. 1).

Also, the results obtained from the low-dose chest CT protocol in the diagnosis of COVID-19 pneumonia [68, 69] were in agreement with low-dose chest CT protocols used in detecting other diseases (for example, in the identification of pulmonary nodules, in lung cancer screening, etc.) [61, 63], as the findings showed that the radiological features resulted from low-dose protocols can be comparable to those obtained from the standard-dose protocols, with a significant reduction in radiation dose.

## Conclusion

Given the growing number of chest CT scan done during the COVID-19 pandemic, the increased radiation burden on patients and medical staff is becoming a matter of concern. By evaluating the CT scanning parameters used in several studies, one can find the necessity for optimizing these parameters. The findings obtained from the low-dose chest CT protocol (compared to the standard-dose protocol) showed that using this protocol is reliable in the detection of COVID-19 pneumonia in daily practice. Moreover, the low-dose chest CT protocol results in a remarkable reduction (up to 89%) in the radiation dose compared to the standard-dose protocol, not lowering the diagnostic accuracy of COVID-19-induced pneumonia in CT images. Therefore, its employment in the era of the COVID-19 pandemic is highly recommended.

One of the main limitations of using the low-dose chest CT protocols to detect COVID-19 pneumonia is that there are few studies available which have compared the radiological findings in both low-dose and standard-dose protocols; hence, further comparative studies are suggested to get assured of low-dose CT scan sufficiency in diagnosing COVID-19 pneumonia.

## Data Availability

Not applicable.

## Abbreviations

ALARA: As low as reasonably achievable
CT: Computed tomography
COVID-19: Coronavirus 2019
2019-nCoV: 2019 novel coronavirus
CTDI: Computed tomography dose index
DLP: Dose length product
RT-PCR: Reverse transcription-polymerase chain reaction
NCIP: Novel coronavirus-infected pneumonia
LNT: Linear no-threshold
